# The Influence of Internet Use on Perceived Information Credibility Among Adults in Romania

**DOI:** 10.1192/j.eurpsy.2026.11306

**Published:** 2026-07-31

**Authors:** A. Mihai, R. I. Molnar

**Affiliations:** Psychiatry, University of Medicine, Pharmacy, Science and Technology George Emil Palade, Targu-Mures, Romania

## Abstract

**Introduction:**

The primary source of information in the current digital climate is the internet. Adults are subjected to high volume of unverified and misleading information, with no education on how to distinguish right or wrong. Therefore, it becomes essential to train the ability to determine the credibility of given information. The ability to assess information credibility is essential to prevent the spread of misinformation.

**Objectives:**

This study aims to explore the relationship between daily internet use and gullibility among Romanian adults. Moreover, it examines how adults distinguish true or false information in the online environment.

**Methods:**

An online questionnaire was administered to a sample of Romanian adults (N =230) . The questionnaire was designed into 4 parts: (1) demographic and digital behavior data, (2) the Metzger & Flanagin Credibility Scale, (3) the Gullibility Scale (Teunisse et al., 2020), and (4) a true/false paragraph. Data were analyzed using Pearson correlations and multiple linear regression models, as well as SPSS software.

**Results:**

Daily internet use was positively associated with gullibility (r = .32, p < .01) and negatively associated with perceived information credibility (r = –.28, p < .01). Regression analysis showed that time spent online significantly predicted gullibility scores (β = .29, p < .01), even after controlling for demographic factors such as age and education. Participants with higher gullibility were less accurate in identifying false statements in the true/false task (β = –.34, p < .001). Education level moderated the relationship, with individuals holding higher education demonstrating greater accuracy in credibility judgments.

**Conclusions:**

Relying on the internet as the sole source of information should be done with extreme precaution. There is a dire need for interventions designed to develop critical thinking and digital literacy, in order to counter the spread of misinformation.

**Disclosure of Interest:**

None Declared

